# Metabolomics Analysis on the Effect of *Cucumaria frondosa* Tentacles Hydrolysates on Cyclophosphamide-Induced Premature Ovarian Insufficiency

**DOI:** 10.3390/antiox14101245

**Published:** 2025-10-17

**Authors:** Shijia Huang, Wenkui Song, Qiuting Wang, Chuyi Liu, Shunmin Gong, Mingbo Li, Leilei Sun

**Affiliations:** 1 School of Life Sciences, Yantai University, Yantai 264005, China; sjhuang01@s.ytu.edu.cn (S.H.); 1981979471@s.ytu.edu.cn (Q.W.); g18807041394@s.ytu.edu.cn (S.G.); mbli@ytu.edu.cn (M.L.); 2 Guangdong Provincial Key Laboratory of Aquatic Products Processing and Safety, National Research and Development Branch Center for Shellfish Processing (Zhanjiang), Guangdong Provincial Engineering Technology Research Center of Seafood, Guangdong Province Engineering Laboratory for Marine Biological Products, College of Food Science and Technology, Guangdong Ocean University, Zhanjiang 524088, China; songwk@gdou.edu.cn; 3 Marine Biomedical Research Institute of Qingdao, Qingdao 266073, China; liucy@ouc.edu.cn; 4 College of Food Science and Engineering, Ocean University of China, Qingdao 266003, China

**Keywords:** premature ovarian insufficiency, *Cucumaria frondosa* tentacles hydrolysates, oxidative stress, cyclophosphamide, metabolomics

## Abstract

Background: Premature ovarian insufficiency (POI) poses a significant challenge for women. The effects of *Cucumaria frondosa* tentacles hydrolysates (CFTH) on POI remain to be fully elucidated. Purpose: This study aimed to determine whether CFTH exerts a beneficial effect on ovarian function using a POI mouse model and to investigate the underlying mechanisms of action. Methods: In this study, we characterized the amino acid composition and physicochemical properties of CFTH. The POI model was established by administering 100 mg/kg of cyclophosphamide (CP). We assessed the regulation of the estrous cycle, hormone levels, ovarian cell apoptosis, and oxidative stress markers in POI mice. Differences in ovarian and uterine morphology among the different groups were observed. Furthermore, metabolomics analysis was employed to explore potential mechanisms. Results: CFTH treatment reversed the dysregulation of the estrous cycle and hormone levels. TUNEL analysis revealed that CFTH treatment significantly reduced apoptosis in granulosa cells and altered the expression levels of apoptosis-related genes at the mRNA level. Additionally, CFTH significantly increased superoxide dismutase activity and decreased malondialdehyde levels, thereby mitigating oxidative stress. Metabolomics analysis suggested that CFTH may ameliorate ovarian dysfunction by regulating steroid biosynthesis and the cGMP-PKG signaling pathway. Conclusions: These findings suggest that CFTH may serve as an effective strategy for alleviating POI. Further research is warranted to verify the long-term safety and effectiveness of CFTH in humans.

## 1. Introduction

Premature ovarian insufficiency (POI), also referred to as premature ovarian failure (POF), predominantly affects women under the age of 40. Characterized by elevated serum follicle-stimulating hormone (FSH) levels and decreased estrogen (E2), POI can lead to menopause, ovarian failure, and infertility, accompanied by serious complications such as osteoporosis, cardiovascular disease, and autoimmune disorders [1,2]. The precise mechanisms underlying the pathogenesis of POI remain unclear and may involve a variety of factors, including genetic predispositions, metabolic disorders, and iatrogenic influences such as chemotherapy and radiotherapy [3]. Granulosa cells (GCs) play a crucial role in supporting oocyte development and follicle maturation through autocrine and paracrine signaling while regulating gonadotropin levels [4]. Dysfunction of GCs leads to follicular atresia, hormonal imbalance, and diminished ovarian reserve, ultimately resulting in ovarian dysfunction. Moreover, abnormal follicular atresia accelerates the failure of ovarian follicles, further exacerbating ovarian dysfunction [5]. Hormone replacement therapy (HRT) currently represents the primary intervention for POI. However, research indicates that long-term HRT use may elevate the risk of breast cancer, ovarian cancer, and thromboembolic events [6]. Therefore, the advancement of effective treatment strategies is particularly crucial for the prevention and management of POI.

In recent years, dietary supplements have garnered significant attention due to their safety and potential efficacy. Zhang et al. reported that sturgeon swim bladder peptide can protect mice from cyclophosphamide (CP)-induced POI by regulating serum hormone levels and upregulating the Bcl-2/Bax signaling pathway [7]. In addition, Liu et al. found that Sepia esculenta ink polysaccharide significantly mitigates CP-induced ovarian toxicity by activating the Nrf2/ARE signaling pathway [8]. Moreover, it can be concluded that marine by-products exhibit unique potential in improving ovarian function. *Cucumaria frondosa*, commonly known as the sea cucumber (Echinodermata: *Holothurioidea*), is the most prevalent sea cucumber species in the North Atlantic [9]. Research indicates that *Cucumaria frondosa* exhibits multiple functional activities such as anti-inflammatory, antioxidant, anti-fatigue, and anticancer effects [10]. *Cucumaria frondosa* feeds on suspended matter in seawater using its tentacles, which are often regarded as processing waste and remain underutilized. Studies have demonstrated that the tentacles of *Cucumaria frondosa* contain the same nutritional components as the body wall, such as proteins, polysaccharides, lipids, vitamins, and both are valuable sources of flavonoids [11]. Certain flavonoids can help maintain ovarian hormone balance by binding to various types of estrogen receptors, owing to their molecular structure′s similarity to endogenous estrogen and estradiol [12].

*Cucumaria frondosa* tentacles exhibit favorable beneficial effects and have been studied for their non-toxic properties [13]. A previous study demonstrated that sea cucumber hydrolysates effectively reduced follicular atresia and alleviate the symptoms of POI by upregulating the expression of genes related to sex hormone synthesis in the ovary [14]. However, the molecular mechanism underlying the effects of *Cucumaria frondosa* tentacles in the treatment of POI remains unclear. This research evaluated the protective effects of *Cucumaria frondosa* tentacles hydrolysates (CFTH) in a mouse model of POI induced by CP. The evaluation included assessments of body weight and organ index, histological analyses, observation of the estrous cycle, serum hormone levels, TUNEL analysis, apoptosis gene expression, and oxidative stress markers. Finally, the potential mechanism of action of CFTH was analyzed through metabolomics.

## 2. Materials and Methods

### 2.1. Materials and Reagents

The *Cucumaria frondosa* tentacles were procured from the Yantai Seafood Market. They were cleaned with deionized water, freeze-dried for 48 h, crushed into a powder, and stored in a desiccator for later use. Flavourzyme (15,000 U/g) was obtained from Solarbio Biotechnology Co., Ltd. (Beijing, China). CP and SIF were sourced from Picasso Biochemical Technology Co., Ltd. (Shanghai, China) and Macklin Biochemical Co., Ltd. (Shanghai, China), respectively. All other reagents and chemicals utilized in this study were of analytical grade and were acquired from Sinopharm Chemical Reagent Co., Ltd. (Shanghai, China).

### 2.2. Preparation of CFTH

The enzymatic hydrolysis of the sample was conducted at 40 °C for 9 h, under conditions of a solid–liquid ratio of 1:6 (*w*/*v*), a pH of 5.8, and an enzyme dosage (flavourzyme) of 10,000 U/g. The mixture was immediately boiled for 15 min to inactivate the enzyme, followed by centrifugation at 10,000 rpm for 15 min. The collected supernatant was then freeze-dried to obtain CFTH.

### 2.3. Amino Acid Composition

The amino acid composition of CFTH was determined according to the method described by Liu et al., with slight modifications [15]. CFTH was mixed with 10 mL of 6 M HCl and hydrolyzed at 110 °C for 22 h. The resulting solution was filtered and diluted to 50 mL. Finally, it was filtered through a 0.22 μm membrane and analyzed using an amino acid analyzer (Hitachi, Tokyo, Japan). 

### 2.4. ζ-Potential, Particle Size Distribution, and Polydispersity Index (PDI)

The ζ-potential, particle size distribution, and PDI of the CFTH were measured using a Zeta Sizer Nano-ZS (Nano Brook, Brookhaven, USA), following the method described by Li et al. [16] All measurements were performed in triplicate at a temperature of 25 ± 1 °C.

### 2.5. Animals and Experimental Design

Forty-five female ICR mice, aged 8–10 weeks and weighing 30 ± 2 g, were purchased from Jinan Peng Yue Laboratory Animal Breeding Co., Ltd. During the experiment, mice were able to obtain sufficient drinking water and feed every day. Clean cages and bedding were replaced every 6 days. The laboratory is in a sterile environment and undergoes a 12 h light/dark cycle.

The POI mouse model was established following an initial 7-day adaptation period. Soy isoflavone (SIF) served as a positive control. Mice were randomly assigned to five groups: control, model (POI), positive (SIF), low-dose CFTH intervention (CFTH-L), and high-dose CFTH intervention (CFTH-H), with nine mice in each group. The model, positive, and CFTH intervention groups received daily intraperitoneal injections of 100 mg/kg CP for the first three days, while the control group received an equivalent volume of saline. After three days, mice in the SIF, CFTH-L, and CFTH-H groups were gavaged daily with 400 mg/kg SIF, 200 mg/kg CFTH, and 600 mg/kg CFTH, respectively, for four weeks. Both the control and POI groups were gavaged with equal volumes of saline daily. The body weight of the mice was monitored daily, and vaginal smears were collected during the 2nd and 4th weeks. Following the treatment process, the mice were anesthetized via an intraperitoneal injection of a 1% pentobarbital sodium solution. Euthanasia was performed through cervical dislocation. Blood samples were collected from the mice and centrifuged at 3000× *g* for 10 min to obtain serum for further analysis. The uterus and a portion of the ovarian tissue were preserved at −80 °C for subsequent analysis, while another portion of the ovarian slices was fixed in 4% paraformaldehyde for histological examination.

### 2.6. Observation of the Estrous Cycle

The estrous cycle of mice during the second and fourth weeks was determined through vaginal cell smears. For eight consecutive days, cells from the vaginal orifice of the mice were collected. The specific procedure involved using a cotton swab to collect secretions from the vaginal orifice, followed by dipping the swab in normal saline and smearing the collected secretions onto the center of a microscope slide. After staining with 0.4% methylene blue, the morphology of the cells was observed under a light microscope to ascertain the estrus stage of the mice [17].

### 2.7. Organ Index and Area of the Ovary and Uterus

At the conclusion of the experiment, the ovaries and uteri of the mice were harvested. Excess fat was removed, and the tissues were weighed and photographed. The areas of the ovaries were subsequently calculated using ImageJ software (version 1.53c).

### 2.8. Histological Examination of the Ovary and Uterus

Post-experiment, the ovaries and uteri of the mice were fixed in 4% paraformaldehyde, dehydrated, embedded in paraffin, and sectioned. The sections were then stained with hematoxylin and eosin (H&E) and rinsed with distilled water. The morphology of the ovarian and uterine tissue was observed under an optical microscope. The number of follicles was subsequently quantified using ImageJ software (version 1.53c).

### 2.9. Determination of Biochemical Parameters

Blood samples from the mice were collected and immediately centrifuged, retaining the upper serum layer for testing. The serum concentrations of testosterone (T), FSH, E2, luteinizing hormone (LH), and anti-Müllerian hormone (AMH) were measured using ELISA kits (Aikang Biomedical R&D Co., Ltd., Zhangjiagang, China) in accordance with the manufacturer’s instructions.

### 2.10. TUNEL

The apoptosis of mouse ovarian cells was assessed using terminal deoxynucleotidyl transferase (TdT)-mediated deoxyuridine triphosphate (dUTP) nick end-labeling (TUNEL) analysis [18]. TUNEL-positive cells were visualized using a fluorescence microscope (Leica, Wetzlar, Germany), and the apoptosis rate was calculated using ImageJ software (version 1.53c).

### 2.11. Reverse Transcription-Quantitative PCR (RT-qPCR)

Total RNA was isolated from ovarian tissue using *RNAiso* Plus. Reverse transcription was performed following the instructions provided in the PrimeScript RT kit (TaKaRa, Kusatsu, Japan). Subsequently, the HieffTM qPCR SYBR Green Master Mix (No Rox) kit (YEASEN, Shanghai, China) was utilized for real-time quantitative PCR. In addition, the relative expression levels of mRNA were calculated using the 2^−ΔΔCt^ method. The following primers were employed: Bcl-2 sense: 5′-ACACCCCCTCCTCCAATACT-3′, antisense: 5′-CGCTAGGTGACCCCATTCTT-3′; Bax sense: 5′-CTTTCCTCCTCTCTCCCCCA-3′, antisense: 5′-CACTCGCTCAGCTTCTTGGT-3′; Caspase-3 sense: 5′-GAGCTTGGAACGGTACGCTA-3′, antisense: 5′-GAGTCCACTGACTTGCTCCC-3′.

### 2.12. Serum Oxidative Stress

Serum levels of superoxide dismutase (SOD) and malondialdehyde (MDA) were measured using a standard kit from Nanjing Jiancheng Bioengineering Institute (Nanjing, China).

### 2.13. Non-Targeted Metabolomics

A total of 100 μL of serum sample was mixed with 400 μL of extraction solution (MeOH: ACN, 1:1 (*v*/*v*)). The mixture was vortexed thoroughly, followed by 10 min of ultrasonic treatment and incubation at −40 °C for 1 h. Subsequently, the mixture was centrifuged at 12,000 rpm for 15 min at 4 °C, and the supernatant was transferred to prepare a quality control (QC) sample. The chemical analysis of serum samples was performed using a Vanquish (Thermo Fisher Scientific, Waltham, MA, USA) ultra-high-performance liquid chromatogram (UHPLC) equipped with a Waters ACQUITY UPLC BEH Amide column (Milford, MA, USA, 2.1 mm × 50 mm, 1.7 μm). The mobile phases A consisted of 25 mmol/L ammonium acetate and 25 mmol/L ammonia, while phase B was acetonitrile. Data processing was performed via the Personalbio GenesCloud platform (https://www.genescloud.cn (accessed on 10 February 2025)).

### 2.14. Statistical Analysis

Statistical analyses were conducted using GraphPad Prism 9.0 software, and results were expressed as mean ± SD. One-way ANOVA was employed for group comparisons, with significant differences reported as *p* < 0.05.

## 3. Results

### 3.1. Amino Acid Composition of CFTH

The analysis of the amino acid composition of CFTH prepared using flavourzyme was presented in Table 1. Rigorous analysis of the acid composition derived from CFTH revealed that hydrophobic amino acids constituted 16.894% of the overall composition. Previous studies have demonstrated that hydrophobic amino acids significantly enhance antioxidant activity, particularly in scavenging 2,2-diphenyl-1-picrylhydrazine (DPPH) and H_2_O_2_ free radicals [19].

### 3.2. ζ-Potential, Particle Size Distribution, and PDI of CFTH

The particle size distribution pattern of CFTH is a critical factor influencing its absorption characteristics. The measured particle size distribution of CFTH was found to be 434.20 ± 5.75 nm (Table 2). The PDI serves as an important metric for evaluating the dispersibility of macromolecular polymers. A lower PDI value indicates improved dispersion in water [20]. The ζ-potential provides insight into the surface charge of the particles, with higher absolute values signifying enhanced stability and resistance to aggregation. The ζ-potential of CFTH was measured to exceed −30 mV, indicating its excellent stability.

### 3.3. CFTH Improved the Reproductive Phenotype of POI Mice

**Figure 1:**
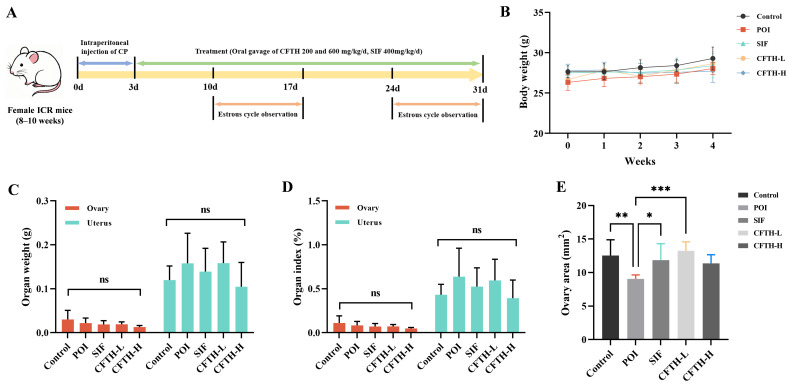
Experimental protocols, body weights, and organ indices in mice. (**A**) Schematic diagram of mouse treatment with SIF and CFTH. (**B**) Dynamic changes in body weight among CFTH-treated mice at varying doses. (**C**) Weights of the ovary and uterus across different groups; (**D**) Ovarian and uterine indices among the various groups; (**E**) Ovary area measurements in mice (*n* = 6). Statistical significance is indicated as follows: * *p* < 0.05, ** *p* < 0.01, *** *p* < 0.001, and ns = no significant difference (*p* > 0.05).

The estrous cycle of female mice, lasting 4 to 5 days, resembles the menstrual cycle in human females but is characterized by a shorter duration. Concurrently, the fluctuations in hormone levels and the sequence of follicle development are highly similar to those observed in human females, thereby facilitating the monitoring of FSH, E2, and AMH levels. Consequently, female mice serve as an ideal model for constructing models of POI. In contrast, alternative approaches, such as in vitro cell models, lack systemic factors, including hormone regulation, immune system interactions, and blood flow. This deficiency makes it challenging to fully replicate the overall pathology of POI. CP is a classic drug utilized to establish POI models, inducing follicle apoptosis through direct DNA damage, reactive oxygen species (ROS) generation, and subsequent ovarian damage [21]. SIF, a commercially available medication recognized for its efficacy in ameliorating the POI phenomenon, significantly up-regulated hormone levels, achieving results comparable to those of the control group [22]. In this study, body weight, organ weight, organ index, and ovarian area were critical indicators for assessing the safety of CFTH. We observed that the body weight of mice in the CP-treated group was lower than that of untreated control mice or those treated with 200 or 600 mg/kg CFTH at the conclusion of the dosing period (Figure 1B). At the end of the four-week trial, CFTH did not significantly impact the weights of the ovaries and uterus, nor their respective indices in POI mice, with the SIF group exhibiting similar outcomes (Figure 1C,D). Furthermore, CFTH-L notably improved the reduced ovarian area characteristic of POI mice (*p* < 0.001), demonstrating effects akin to those observed in the control and SIF groups (Figure 1E).

### 3.4. Estrous Cycle

**Figure 2:**
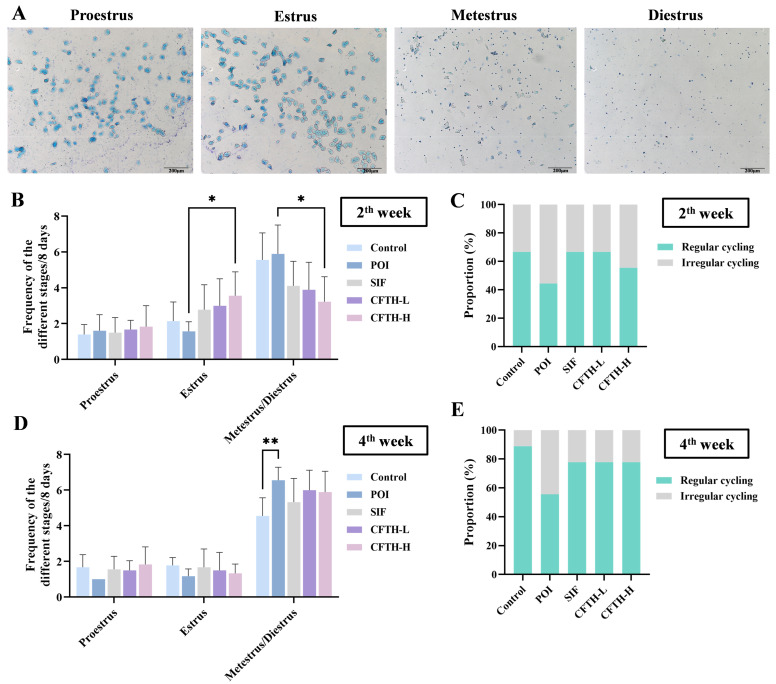
Improvement in estrus cycle by CFTH in POI mice. (**A**) Vaginal cell smears at different stages of estrus; (**B**) Estrous cycle of mice in the second week; (**C**) Estrous cycle proportions in the second week; (**D**) Estrous cycle of mice in the fourth week; (**E**) Estrous cycle proportions of mice in the fourth week (*n* = 6). Statistical significance is indicated as follows: * *p* < 0.05 and ** *p* < 0.01.

At this stage of the experiment, we recorded the progression of the estrous cycle in mice during the second and fourth weeks by observing the cell types and proportions at different stages. The vaginal cell smears indicated significant differences among the mice at various estrous stages [23]. Proestrus was characterized by a predominance of nuclear epithelial cells, accompanied by some keratinized epithelial cells. The estrus stage was predominantly composed of anucleate keratinized epithelial cells, a typical indicator of this phase. The metestrus stage was marked by the presence of both cell types, along with leukocytes. In contrast, the diestrus stage exhibited a high number of leukocytes (Figure 2A).

During the second week, mice in the POI group exhibited a disrupted estrous cycle, while both the SIF and CFTH-L groups showed similar effects as the control group. At this time, the CFTH-H group also demonstrated slight improvement in this phenomenon (Figure 2B,C). By the fourth week, there was a significant increase in the percentage of regular estrous cycles in the CFTH-L and CFTH-H groups compared to previous recordings, effectively mitigating the adverse effects of POI. For stabilized estrus, CFTH displayed comparable effects to the positive drug SIF (Figure 2D,E).

### 3.5. Effects of CFTH on the Morphology of Mice Ovary and Uterus

**Figure 3:**
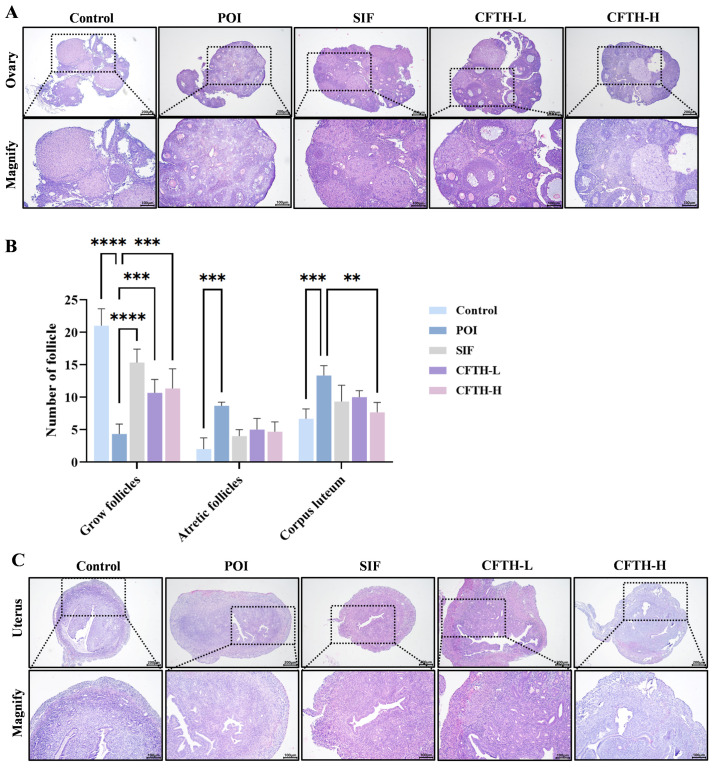
Improvement in ovarian and uterine defects by CFTH in POI mice. (**A**) Ovarian histology of mice; (**B**) Number of follicles at different developmental stages (*n* = 6); (**C**) Uterine histology of mice. Statistical significance is indicated as follows: ** *p* < 0.01, *** *p* < 0.001, and **** *p* < 0.0001.

The development of ovarian follicles is a dynamic regulatory process that reflects ovarian health, and abnormalities in this process can lead to pathological conditions. Follicular atresia refers to the degeneration of follicles during their growth and development [24]. Compared to the control group, the number of growing follicles in the POI group induced by CP decreased, while the number of atretic follicles increased, and antral follicle development was impaired. Notably, both the low-dose and high-dose CFTH groups exhibited a significant reduction in the number of blocked follicles compared to the POI group (Figure 3A,B). This finding is further corroborated by measurements of serum sex hormones. Additionally, the POI group exhibited uterine atrophy and decreased stromal cell density compared to the control group, which may be related to changes in sex hormone levels induced by CP. Following CFTH treatment, the uterine morphology showed significant recovery, approaching that of the SIF group (Figure 3C).

### 3.6. Sex Hormones Analysis

**Figure 4:**
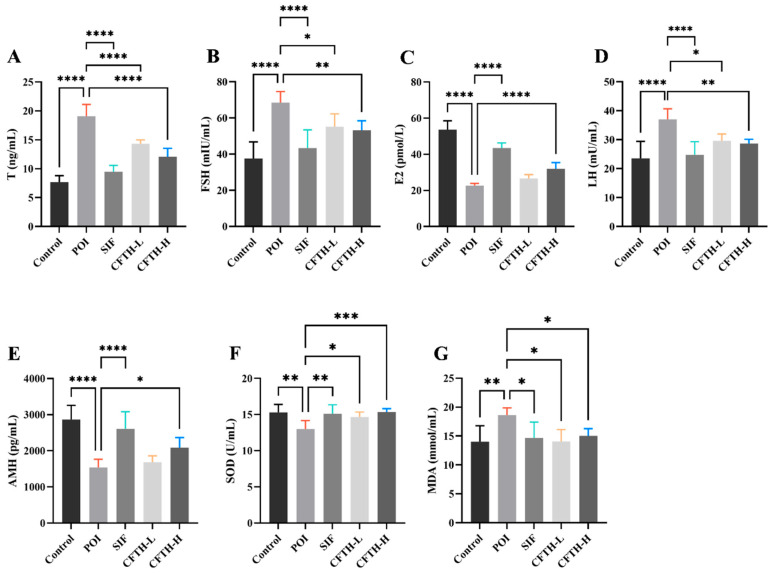
Effect of CFTH on serum sex hormones and oxidative stress in POI mice. (**A**) T; (**B**) FSH; (**C**) E2; (**D**) LH; (**E**) AMH; (**F**) SOD; (**G**) MDA (*n* = 6). Statistical significance is indicated as follows: * *p* < 0.05, ** *p* < 0.01, *** *p* < 0.001, and **** *p* < 0.0001.

Previous studies have demonstrated that POI is associated with decreased levels of E2 and AMH in serum, alongside increased levels of LH, FSH, and T [25,26]. Consequently, we measured the levels of sex hormones across different groups (Figure 4A–E). The results indicated that hormone levels in the POI group differed significantly from those in the control group, potentially reflecting ovarian insufficiency. It can be concluded that CFTH-L significantly downregulated the levels of T, FSH, and LH, although it did not exert a significant effect on E2 and AMH levels. Furthermore, the CFTH-H group demonstrated a more favorable effect in regulating hormone levels compared to the low-dose group. CFTH reversed the ovarian hormone disorder induced by CP, which aligns with the findings of Miao et al. [27].

### 3.7. Effect of CFTH on Oxidative Stress

ROS play a crucial role in regulating follicle growth and sex hormone synthesis. However, when the balance of ROS is disrupted, it can induce oxidative stress and damage oocytes [28]. SOD is one of the most important antioxidant enzymes in the body, and its expression level increases in response to oxidative stress. Changes in MDA levels correspond to SOD activity [29,30]. The induction of CP increases ROS generation, disrupts the normal function of granulosa cells, and leads to ovarian aging [31]. In this work, SOD and MDA levels were further determined in mice. In the POI model, ROS accumulation leads to membrane damage in ovarian cells, resulting in a significant increase in MDA levels (*p* < 0.01). The CFTH-L and CFTH-H groups significantly increased SOD activity and scavenging of ROS, thereby reversing this phenomenon (Figure 4F,G).

### 3.8. TUNEL and RT-qPCR Analysis

**Figure 5:**
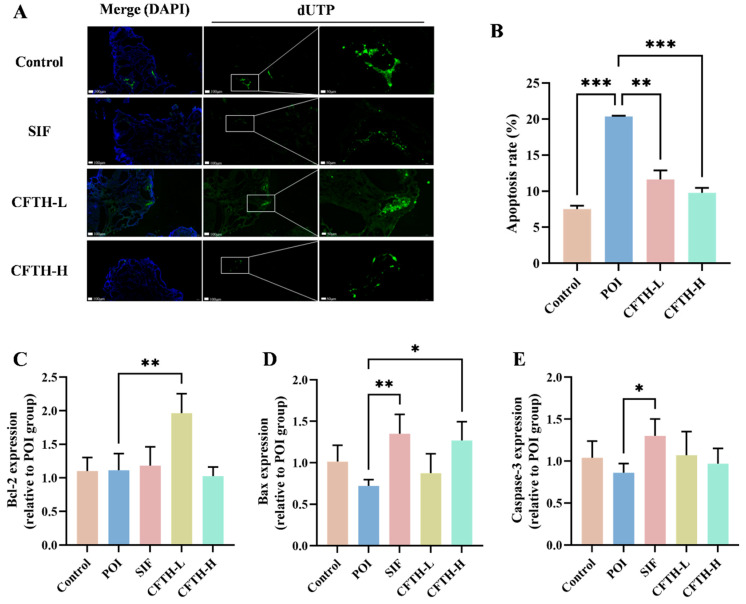
CFTH reduces apoptosis of mice ovarian GCs by regulating the expression level of apoptotic genes. (**A**) TUNEL staining images of ovaries. Nuclei stained with DAPI are represented in blue, while apoptotic cells are indicated in green; (**B**) Quantification of apoptosis in pre-antral and antral follicles, with apoptosis rate expressed as apoptotic/total follicles (*n* = 6); (**C**) Expression of Bcl-2; (**D**) Expression of Bax; (**E**) Expression of Caspase-3. Statistical significance is indicated as follows: * *p* < 0.05, ** *p* < 0.01, and *** *p* < 0.001.

TUNEL fluorescence staining serves as a reliable indicator of apoptosis in ovarian cells, a critical factor contributing to diminished ovarian reserve function and follicular atresia [32]. Compared to the control group, the rate of apoptosis in ovarian GCs in the POI group was significantly elevated (*p* < 0.01). Notably, the CFTH-L and CFTH-H groups demonstrated a significant downregulation of this apoptosis rate (Figure 5A,B). Furthermore, Xu et al. suggested that the therapeutic efficacy of the sample on POI mice may be modulated by apoptosis-related proteins, including Bcl-2, Bax, and caspase-3, present in the ovaries [33]. Additionally, the expression levels of apoptosis-related genes in ovarian cells were assessed. The findings revealed that low-dose CFTH significantly increased Bcl-2 expression and inhibited cell apoptosis (*p* < 0.01), whereas high-dose CFTH significantly elevated Bax expression (*p* < 0.05) (Figure 5C–E). Overall, CFTH effectively reduced the apoptosis rate of GCs in mouse ovaries by inhibiting the apoptotic pathway.

### 3.9. Non-Targeted Metabolomics Analysis

**Figure 6:**
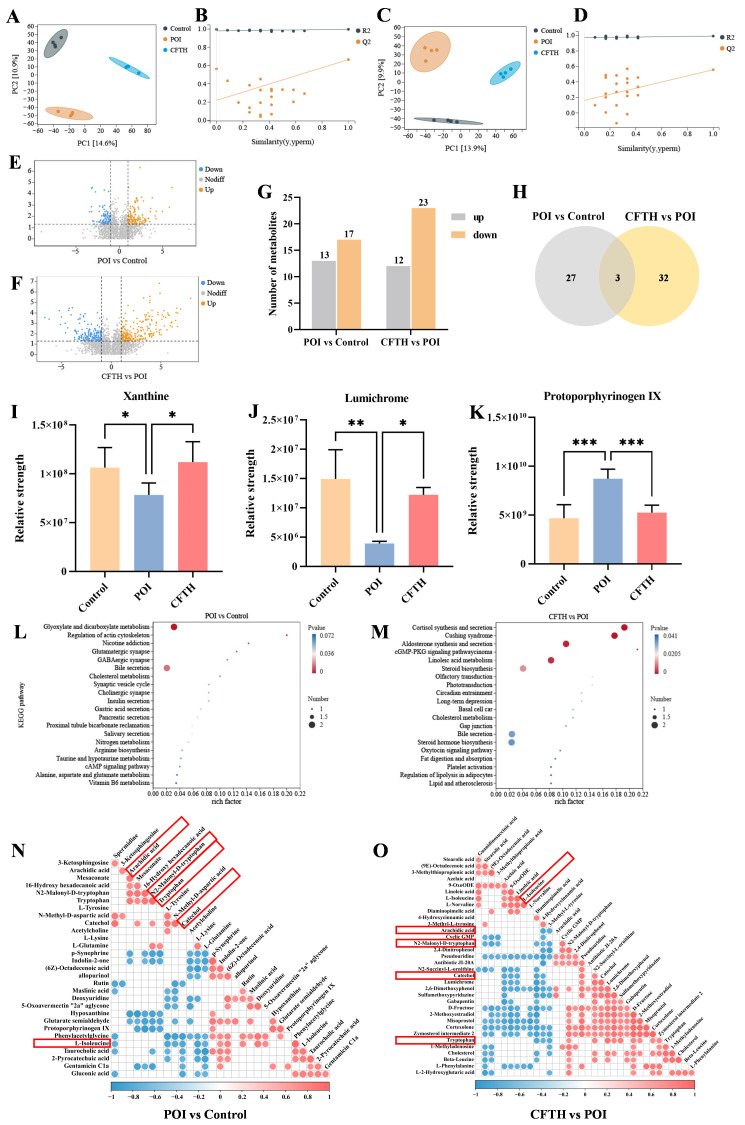
Metabolomic analysis of serum samples obtained from POI mice treated with CFTH. (**A**,**B**) Positive ion modeling PLS-DA and displacement test. (**C**,**D**) Negative ion modeling PLS-DA and displacement test (*n* = 4). (**E**,**F**) Volcanic plot of the metabolomics data (POI vs. Control and CFTH vs. POI). (**G**) Bar chart of differential metabolite screening; (**H**) Venn diagram (POI vs. Control and CFTH vs. POI). (**I**–**K**) Common differential metabolites. (**L**,**M**) KEGG enrichment analyses of the differentially enriched pathways (POI vs. Control and CFTH vs. POI). (**N**,**O**) Pearson correlation analysis of differential metabolites (POI vs. Control and CFTH vs. POI). Statistical significance is indicated as follows: * *p* < 0.05, ** *p* < 0.01, and *** *p* < 0.001.

We conducted a non-targeted metabolomics analysis of serum metabolic profiles using UHPLC-Q-TOF-MS/MS to investigate the mechanism of action of CFTH in alleviating POI. The results from the partial least squares discriminant analysis (PLS-DA) score plot demonstrated that the quality control samples were well-clustered in both positive and negative ion modes, indicating that the analytical method exhibited good stability and reliability, making it suitable for subsequent analysis of serum metabolites. To confirm the reliability of the model, a permutation test was performed, revealing that in both positive and negative ion modes, the R^2^ value exceeded 0.5 while Q^2^ was negative, suggesting that the models were not over-fitted and could accurately reflect the real changes in metabolites within the samples (Figure 6A–D) [34]. Compared to the control group, 17 metabolites downregulated and 13 metabolites upregulated in the POI group, including dimethylglycine, indolin-2-one, per-ethanol, hydroxytryptophan, 5-hydroxyindoleacetic acid, and octadecenoic acid. Notably, p-adrenaline, protoporphyrinogen IX, and glutaric acid semialdehyde were significantly elevated in the POI group, whereas xanthine and lumichrome showed significant decreases (Figure 6E,F). Figure 6G illustrated a certain degree of variation in foreign body levels in the CP-induced POI group, while the CFTH treatment altered these results to some extent. Three key metabolites were identified based on the criteria of variable importance in projection (VIP) > 1, fold change > 2 or < 0.05, and *p* < 0.05 (Figure 6H) [35].

The differential metabolites identified from the aforementioned experiments included xanthine, lumichrome, and protoporphyrinogen IX. The variations in metabolite quantities among different groups were illustrated in Figure 6I–K. Compared to the control group, the POI group exhibited a significant decrease in xanthine and lumichrome levels, alongside a significant increase in protoporphyrin IX levels (*p* < 0.001). Notably, CFTH treatment significantly reversed these changes. Massaro et al. [36] reported that lumichrome functioned as a bioactive factor with antioxidant properties. This increase in lumichrome levels post-CFTH treatment may enhance the antioxidant capacity of ovarian cells, thereby preserving the stability of the follicular microenvironment. Both xanthin and protoporphyrin IX, as endogenous metabolites, can adversely affect health if their levels are chronically imbalanced. Xanthine serves as a crucial intermediate in purine metabolism, and its deficiency in POI mice leads to abnormal purine metabolism, which in turn affects oocyte maturation and hormone synthesis. CFTH treatment promotes xanthine production, restores normal purine metabolism, and alleviates POI. Du et al. [37] indicated that a high intake of xanthine may reduce the risk of ovarian cancer. Ovarian tissue is particularly susceptible to oxidative damage, with excessive accumulation of ROS potentially leading to direct damage to oocytes and granulosa cells, thereby accelerating follicular atresia [28]. Protoporphyrin IX is a vital intermediate in the heme synthesis pathway. Its abnormal accumulation may result in excessive ROS production, potentially damaging oocytes. This phenomenon may be associated with insufficient FAD cofactors caused by riboflavin deficiency [38]. This study found that post-CFTH treatment, serum levels of xanthine and lumichrome in POI mice were upregulated, while protoporphyrin IX levels were downregulated. This suggests a potential pathway through which these compounds may offer protection against POI.

To elucidate the protective mechanism of CFTH on POI, this study supplemented it with a metabolic pathway enrichment analysis. The results indicated that CP, as a chemotherapy drug, can induce metabolic disorders in mice by inducing hormonal imbalance and oxidative stress. This specifically involves significant alterations in pathways such as glyoxylate and dicarboxylate metabolism, regulation of the actin cytoskeleton, glutamatergic synapse, GABAergic synapse, and bile secretion (Figure 6L). The interactions among these pathways collectively lead to ovarian cell damage, hormone imbalance, and follicle depletion, ultimately resulting in POI. Following CFTH treatment, significant changes were observed in metabolic pathways related to steroid biosynthesis, cortisol synthesis and secretion, aldosterone synthesis and secretion, the cGMP-PKG signaling pathway, and linoleic acid (LA) metabolism (Figure 6M). By regulating these pathways through multiple targets, it is possible to synergistically enhance ovarian hormone secretion and mitigate oxidative damage. Furthermore, we validated our hypothesis using Pearson correlation analysis of differential metabolites, with results illustrated in Figure 6N,O. Following CFTH treatment, the metabolite L-isoleucine was found to be upregulated, whereas arachidic acid, trypsin, N2-Mlonyl-D-tryptophan, and catechol were downregulated. Di et al. [39] demonstrated that isoleucine can inhibit the apoptosis-promoting effects induced by dehydroepiandrosterone, enhance cell proliferation, and restore mitochondrial function. The downregulation of specific metabolites, such as arachidic acid, trypsin, N2-Mlonyl-D-tryptophan, and catechol, contributes to the restoration of normal metabolism in POI mice.

## 4. Discussion

*Cucumaria frondosa* has been demonstrated to exhibit multiple functional activities, including anti-aging, anti-fatigue, anti-inflammatory, hypoglycemic, and antibacterial effects [40]. However, tentacles are frequently discarded as a byproduct during the processing of *Cucumaria frondosa*. This study employed enzymatic hydrolysis to prepare CFTH, thereby enhancing the utilization of *Cucumaria frondosa* tentacles. By comparing body weight and organ indices, observing the estrous cycle, and conducting pathological examinations, it was concluded that POI mice treated with CFTH experienced reversal of weight loss, an increase in ovarian area, normalization of the estrous cycle, and a reduction in the number of atretic follicles. Meng et al. [41] reported similar findings after administering quercetin to POI mice, noting improvements in body weight, ovarian index, and a decrease in the number of atretic follicles. Furthermore, analyses of hormone levels, oxidative stress, TUNEL assay, and expression of apoptosis-related genes revealed that both high and low doses of CFTH could stabilize hormone levels, decrease the apoptosis rate of GCs, and mitigate oxidative stress. These findings suggest that CFTH can effectively ameliorate ovarian dysfunction, consistent with the results reported by Li et al. [42]. Finally, metabolomics analysis was conducted to evaluate the changes in metabolic pathway enrichment function in POI mice following CFTH treatment.

Hormone replacement therapy (HRT) is regarded as an effective treatment for POI due to its capacity to alleviate vasoconstriction and prevent bone loss and cardiovascular diseases. Specifically, steroid hormones, including estrogen and progesterone, are utilized to compensate for the deficiency of sex hormones in the body [43]. These steroid hormones are predominantly generated by the ovaries and are intricately related to estrogen synthesis. The biosynthesis of steroid hormones directly influences the normal functioning of the ovaries [44]. Research indicates that patients with ovarian dysfunction are typically characterized by elevated nocturnal cortisol levels. This cortisol imbalance is significantly associated with complications such as fatigue and vegetative depression [45]. Cortisol and aldosterone, both steroid hormones secreted by the adrenal cortex, serve as crucial regulators of metabolic function. Cortisol regulates the hypothalamic-pituitary-ovarian (HPO) axis, the central network of the female reproductive system, and plays a crucial role in follicular development [46]. Moreover, Tian et al. demonstrated that nitrous oxide enhances glucose uptake by GCs through the activation of the cGMP-PKG pathway, thereby facilitating granulocyte development [47]. This process is accompanied by the absorption and utilization of glucose and amino acid metabolites, ultimately promoting cellular renewal and proliferation [48]. The metabolic dysregulation of aldosterone synthesis and secretion is closely linked to the onset of polycystic ovary syndrome (PCOS), a prevalent condition among women. Notably, Armanini et al. found that abnormal elevations in aldosterone can exacerbate the inflammatory state of the ovaries [49]. In addition, there is currently no positive correlation between LA intake and the incidence of POI. Rather, increased levels of LA in tissues are associated with a reduced incidence of cardiovascular diseases, which is one of the complications associated with POI [50].

In summary, CFTH synergistically regulates various pathways, including steroid hormone biosynthesis (especially cortisol and aldosterone) and the cGMP-PKG signaling pathway, to restore metabolic homeostasis in ovarian cells and improve POI. Additionally, it reduces the risk of complications associated with POI, such as cardiovascular disease, by modulating LA metabolism.

## 5. Conclusions

The existing data indicated that CFTH, prepared through enzymatic hydrolysis, significantly reversed CP-induced POI primarily by repairing follicular atresia, restoring ovarian reserve, and inhibiting GCs apoptosis. Metabolomics analysis revealed that CFTH mainly ameliorated endocrine disorders by regulating steroid synthesis pathways, thereby restoring ovarian function in POI mice. Additionally, xanthine and protoporphyrin IX may serve as potential biomarkers for monitoring the disease status of POI. However, several limitations must be noted. Firstly, while CP-induced POI mouse models are widely used, they may not fully account for the etiology of human POI, which can arise from multiple factors, including autoimmune diseases, gene mutations, and environmental influences. Secondly, although we have demonstrated that CFTH can upregulate the steroid pathway in POI mice, the precise molecular mechanisms need to be fully elucidated. Further research is necessary to identify the targets and pathways of action between the two. Finally, it is essential to evaluate the long-term safety and efficacy of CFTH treatment, particularly in clinical settings. This research enhances the understanding of CFTH’s function, promotes the high-value utilization of marine by-products, and provides new insights into the development of natural functional foods aimed at alleviating POI.

## Figures and Tables

**Table 1:** The amino acid composition of CFTH.

Amino Acid	Ratio (g/100 g)
Asp	3.096
Thr	1.428
Ser	1.785
Glu	5.098
Gly	4.925
Ala	2.336
Cys	0.000
Val	1.821
Met	0.902
Ile	1.398
Leu	1.577
Tyr	0.658
Phe	1.011
Lys	2.034
His	0.849
Arg	2.268
Pro	2.266
Total amino acids	33.452
Essential amino acids ^1^	7.126
Hydrophobic amino acids ^2^	16.894
Negatively charged amino acids ^3^	8.167
Positively charged amino acids ^4^	5.151
Aromatic amino acids ^5^	2.518

^1^ Essential amino acids = Thr, Met, Val, Ile, Leu, and Trp; ^2^ Hydrophobic amino acids = Ala, Val, Ile, Leu, Tyr, Phe, Trp, and Met; ^3^ Negatively charged amino acids = asx (asparagine and aspartic acid) and glx (glutamine and glutamic acid); ^4^ Positively charged amino acids = Arg, His, and Lys; ^5^ Aromatic amino acids = Phe, Trp, Tyr, and His.

**Table 2:** ζ-Potential, particle size distribution, and PDI of CFTH.

	ζ-Potential (mV)	Particle Size Distribution (nm)	PDI
CFTH	−34.28 ± 1.70	434.20 ± 5.75	0.32 ± 0.01

## Data Availability

The original contributions presented in this study are included in the article. Further inquiries can be directed to the corresponding author.

## Abbreviations

The following abbreviations are used in this manuscript:

CFTH	*Cucumaria frondosa* tentacles hydrolysates
POI	Premature ovarian insufficiency
POF	Premature ovarian failure
CP	Cyclophosphamide
GCs	Granulosa cells
HRT	Hormone replacement therapy
SIF	Soy isoflavone
CFTH-L	Low-dose CFTH intervention
CFTH-H	High-dose CFTH intervention
PDI	Polydispersity index
Nrf2	Nuclear factor erythroid 2-related factor 2
ARE	Antioxidant response element
Bcl-2	B-cell lymphoma-2
Bax	BCL2-Associated X
Caspase-3	Cysteine aspartate specific protease 3
DPPH	2,2-diphenyl-1-picrylhydrazine
SOD	Superoxide dismutase
MDA	Malondialdehyde
H&E	Hematoxylin and eosin
T	Testosterone
FSH	Follicle-Stimulating Hormone
E2	Estradiol
LH	Luteinizing hormone
AMH	Anti-Müllerian hormone
TdT	Terminal deoxynucleotidyl transferase
dUTP	Deoxyuridine triphosphate
TUNEL	TdT-mediated dUTP nick end labeling
RT-qPCR	Reverse transcription-quantitative polymerase chain reaction
UHPLC	Ultra-high-performance liquid chromatogram
PLS-DA	Partial least squares discriminant analysis
FAD	Flavin Adenine Dinucleotide
HPO	Hypothalamic-pituitary-ovarian
PCOS	Polycystic ovary syndrome
